# Seasonal variation of health in Asian elephants

**DOI:** 10.1093/conphys/coaa119

**Published:** 2020-12-29

**Authors:** Diogo J Franco dos Santos, Vérane Berger, Robin Cristofari, Win Htut, U Kyaw Nyein, Htoo Htoo Aung, Sophie Reichert, Virpi Lummaa

**Affiliations:** 1 Department of Biology, University of Turku, Yliopistonmäki (Vesilinnantie 5) 20014 Turku, Finland; 2 Myanma Timber Enterprise, Ministry of Natural Resources and Environmental Conservation, West Gyogone Forest Compound, Bayint Naung Road, 11011 Insein Township, Yangon, Myanmar

**Keywords:** Environmental heterogeneity, health markers, long-lived mammals, sub-tropical climate

## Abstract

Long-lived species are often predicted to be buffered against seasonal variation: longevity means low annual mortality and reproductive rates and annual variability in climate may therefore have a smaller impact on population growth rates of long-lived species in comparison to short-lived ones. However, little is known of the physiological mechanisms underlying such patterns in long-lived species. In this study, we investigated seasonal variation in the health of Asian elephants living in a seasonal monsoon climate. We used two complementary methods: (i) global and (ii) trait-by-trait analyses of seasonal effects on 23 health parameters of 225 individually marked elephants with known age and reproductive and health history, with repeated measures per individual over a 26-month period. The global analysis highlighted the biggest differences in health between the hot and monsoon seasons. Our trait-specific analyses identified the physiological functions underlying such health variation in different ecological settings, including haematological, immunological, muscular, kidney and liver functions, as well as protein balance and electrolytes. Overall, the results suggest that even long-lived, large mammals may experience physiological changes in response to seasonal variation that in extreme circumstances can pose a significant health risk.

## Teaser

Environmental seasonality in a sub-tropical monsoon climate influences the physiological measures of health in a long-lived mammal, the Asian elephant. There is a marked difference between the hot and monsoon seasons across a wide range of physiological functions and systems.

## Introduction

In nature, temporal variation in environmental conditions is ubiquitous, introducing individual differences in life history traits and fitness within populations (Behrman *et al.*, 2015). Some of this environmental variation is predictable within a year and is driven by temporal variation in abiotic and biotic factors across the different seasons (Behrman *et al.*, 2015). Accumulating evidence across a range of taxa now documents how seasonal variability affects key factors such as food availability (Richards and Windsor, 2007), infectious disease exposure (Dowell, 2001; Grassly and Fraser, 2006), body mass (Perret and Aujard, 2001; Albon *et al.*, 2016), reproduction (Perret and Aujard, 2001; Verhulst and Nilsson, 2008) and survival (Schaub and Vaterlaus-Schlegel, 2001; Mumby *et al.*, 2013a). However, far less is known of the underlying physiological mechanisms between environmental variation and health. Seasonality is responsible for changes in temperature, rainfall and other weather patterns, as well as food availability and disease pattern (Cavalheri *et al.*, 2018). Understanding how these multiple interacting factors affect physiological markers of health—which ultimately lead to reduced (or increased) growth, reproduction and survival of individuals—is essential to the development of better conservation strategies in endangered species.

One approach to study the effects of environmental variation on physiological markers of health in long-lived species is to monitor species inhabiting seasonal environments and observe longitudinally how their different health parameters respond to the weather fluctuations and the accompanying environmental changes (Albon *et al.*, 2016). Seasonal variation is known to affect health (i.e. correct performance of physiological body functions that ensure homeostasis; Gunnarsson, 2006) in many species across different taxa living in a variety of environments, including mammals (grey wolves: Butler *et al.*, 2006; harbour seals: Trumble *et al.*, 2006; beluga whales: Norman *et al.*, 2012), birds (Eurasian skylarks: Hegemann *et al.*, 2012) and reptiles (yellow-marginated box turtle: Yu *et al.*, 2014). Most studies, however, have focused on animals that live at high latitudes with significant differences in temperature and rainfall, as well as in photoperiodicity between summer and winter, or with hibernating mammals such as bears (DelGiudice *et al.*, 1992; Hissa *et al.*, 1994; Butler *et al.*, 2006; Yang *et al.*, 2018). In contrast, a little research exists on long-lived animals exposed to moderate climates and stable photoperiodicity but still with seasonal fluctuations in weather. Moreover, most previous studies have focused on one or two aspects of physiology rather than a more interconnected and broad quantification of health, and they rarely monitor the same individuals longitudinally across different seasons, which limits our ability to understand different responses of the same individuals to ecological challenges.

Asian elephants (*Elephas maximus*) are large, terrestrial mammals with very long lifespans (more than 80 years in some cases) that range in sub-tropical regions with pronounced seasonal variation (Mumby *et al.*, 2013b). In their core range (e.g. India, Myanmar and Thailand), it is possible to distinguish three seasons with marked differences in weather and ecology (Romatschke and Houze, 2011). The hot season from March to June is characterized by high temperatures and no rainfall, the monsoon season from July to October also displays high temperatures but with very high rainfall levels and the cool season from November to February is characterized by lower temperatures and reduced rainfall. Recently, extreme weather patterns during the hot and monsoon seasons are on the rise (Wassmann *et al.*, 2009). Asian elephants are a particularly interesting species to study the link between such environmental variation and health, as they are not seasonal breeders but instead females can conceive at any time of the year during their 16-week oestrus cycle. Nevertheless, their birth rate varies seasonally: being the highest in the cold season and the lowest in the monsoon particularly for the firstborn calves, whose odds of being born between December and March were 3.17 higher than the odds of being born between May and August, although both periods cover 4 months (Mumby *et al.*, 2013a). Similarly, mortality varies seasonally, with the lowest mortality rates occurring during the monsoon season compared with the relatively higher rates of the cold and hot seasons, varying by a factor of 2.0 between the months with the highest and the lowest mortality (Mumby *et al.*, 2015). Asian elephant populations both in captivity and in the wild are becoming increasingly threatened and are classified by the IUCN as endangered (Choudhury *et al.*, 2008). Hence, understanding seasonal variation in the underlying physiological health measures has become paramount to the efforts to conserve the existing populations.

Yet, to date, few studies have assessed how the health of individuals responds longitudinally to seasonal variation, particularly for species found in more tropical regions. High variations in rainfall and temperature are predicted to have both direct and indirect effects on health, by changing food availability and quality, and even the presence and transmission of certain parasites and diseases (Grassly and Fraser, 2006; Albon *et al.*, 2016). For example, it is known that scarcity of water and excessive temperatures can lead to dehydration and heat stroke across many species. This may be detectable in commonly monitored health parameters such as increases in red blood cell count, due to dehydration and increases in blood urea nitrogen (BUN) and creatinine levels in serum, in response to the reduction of systemic fluids (Fowler and Mikota, 2006). Other factors that should be considered are environmental and handling stress. Stress is responsible for changes in leukocyte profile, represented normally by neutrophilia, monocytosis and lymphopenia (Cirule *et al.*, 2012; Grzelak *et al.*, 2017). Stress could affect the health parameters of Asian elephants particularly during the monsoon season since it is the season in which animals present higher glucocorticoid metabolite concentration (Mumby *et al.*, 2015). However, because studies into such ‘ecological medicine’ in natural populations are exceedingly rare, we currently know very little regarding how different individuals cope with ecological challenges over time and across a range of conditions.

In this study, we investigated seasonal variation in 23 health parameters in Asian elephants differing by age and sex in a sub-tropical monsoon climate (Beck *et al.*, 2018). We covered a wide range of physiological functions, such as haematological, immunological, muscular, kidney and liver functions, as well as protein balance and electrolytes, to gain a global understanding of health variation in different ecological settings. In haematology, we evaluated the haematocrit, haemoglobin and white blood cells. Haematocrit and haemoglobin are the measurements of blood haemoglobin concentration and concentration of circulating red blood cell mass, respectively, and both can be affected by seasonal variation in animal nutritional status, food intake and dehydration (DelGiudice *et al.*, 1992; Hellgren *et al.*, 1993). White blood cell evaluation allows the understanding of the immune response of each individual by observing the presence of five leucocytes that have different functions in the immune system: lymphocytes, monocytes, heterophils, eosinophils and basophils. Elephants have heterophils, not neutrophils, due to the presence of eosinophilic-staining granules in these cells; the cytochemical staining properties and functions of elephant heterophils are the same as neutrophils in other mammals (Fowler and Mikota, 2006). Lymphocytes take part in adaptive immunity while monocytes and neutrophils, being phagocytic cells, are involved in the innate response (Cheynel *et al.*, 2017). Eosinophils induce immunity against internal parasites and are part of the inflammatory response (Cheynel *et al.*, 2017), while basophils act as a defence against macroparasites (Karasuyama *et al.*, 2011).

Serum chemistry analysis assessed kidney function (BUN and creatinine), protein concentrations (total proteins consisting of albumin and globulins), liver enzymes [aspartate aminotransferase (AST) and alkaline phosphatase (ALKP)], lipid storage (triglycerides), muscle integrity [creatine kinase (CK)] and total calcium levels. Of these complimentary serum chemistry health parameters, BUN is the end product of protein metabolism and creatinine is the end product of creatine metabolism in the muscles, and they are both excreted from the body by the kidney (Fowler and Mikota, 2006). They can be affected by nutritional status (BUN) or muscle mass (creatinine) that varies according to season (DelGiudice *et al.*, 1992; Butler *et al.*, 2006) or can increase due to dehydration during the hotter seasons (Fowler and Mikota, 2006). Proteins are responsible for several biological processes (such as molecular transport or even immunity; Macrae, 1993) and are expected to increase in the hot season due to dehydration but could also increase due to infectious agents that increase gamma-globulins (Fowler and Mikota, 2006). AST is present in both the liver and the muscles, with ALKP being liver-specific and CK being muscle-specific. Consequently, an increase in AST with CK would suggest muscle damage, especially in seasons entailing loss of muscle mass (Fowler and Mikota, 2006). Since some of the sampled elephants are working, we also expected to observe an increase in these two enzymes during the working season, which takes place during the monsoon and cold seasons (Lahdenperä *et al.*, 2018). Electrolytes are responsible for normal cell function, electrochemical impulses and acid–base balance (Fowler and Mikota, 2006), which may become elevated during the hot seasons due to water deprivation (Fowler and Mikota, 2006). Finally, triglycerides indicate fat storage levels and are expected to increase in the season(s) when nutritional status is better (Fowler and Mikota, 2006; Morfeld *et al.*, 2016).

Our study design allowed longitudinal monitoring of 225 individually marked elephants with known age and reproductive and health history, taking repeated measures per individual across different seasons over a 26-month study period. This study population is part of the world’s largest population of captive Asian elephants (approximately 3000), consisting of government-owned working elephants in timber camps, employed and centrally managed by the Myanma Timber Enterprise (MTE) (Jackson *et al.*, 2019). Although these elephants are managed as draft and transport animals by the MTE, they are considered ‘semi-captive’ and live largely under natural conditions. They are released in the surrounding forests outside working hours and at night to forage and socialize with wild and other captive conspecifics, and breeding rates are not managed by humans, with mating and birth occurring independently in the forests. MTE elephants benefit from veterinary checks twice a month, but usually only limited treatments were available during the study period. Hence, the population offers a unique opportunity to collect longitudinal measures of the health of individually marked animals with known age, breeding history and veterinary records, but that exhibit foraging, lifespan and breeding patterns comparable with wild elephants (Lahdenperä *et al.*, 2018). Given the seasonally varying mortality and fertility rates in the population (Mumby *et al.*, 2013a,b), we expect that season will have a strong effect on the health parameters and that our study will contribute towards creating a better health management system for this endangered species. However, our study does not focus on the individual clinical perspective but rather on the population health regarding additive changes in the full range of the studied parameters.

## Materials and methods

### Study population

Our study population of government-owned MTE elephants inhabits forests and logging camps distributed across Myanmar. Captive-born timber elephants that comprise most of the current population (Lahdenperä *et al.*, 2018; Jackson *et al.*, 2019) are raised by their mothers and allomothers (Lynch *et al.*, 2019). Pregnant females are given rest from work mid-pregnancy (11 months) until their calf reaches 1 year of age, and after that, mothers continue light work until their calf is 4 years old and capable of foraging independently. At age 4 or 5 years, calves are separated from mothers and are tamed (Min Oo, 2010; Crawley *et al.*, 2020). At the end of this process, they are assigned a rider (mahout), a name, a logbook and a registration number that is marked on their haunches. From age 5–17 years, the elephants are used in light work only, such as transportation, and from 18 years, they are employed to drag logs until their retirement at age 55 years. During retirement, the elephants are held in nursing camps and cared for until their death.

The logbooks of each elephant are also maintained until death, providing our study with vital information on the demography and veterinarian care for every MTE elephant, such as sex, date of birth and date of last medical treatment. The logbooks include individual-specific information including identification number and name, birth origin (captive-born or wild-caught), date of birth, most recent location, mother’s identification number and name (if known), year and place of capture (if wild-captured), year or age of taming, date of death or last known date alive and cause of death. Moreover, the elephants receive twice a month checks by trained veterinarians throughout their lifetime, who closely monitor and record any changes in the elephant’s body condition, health and specific illnesses.

The working season of MTE elephants lasts from mid-June until mid-February, i.e. during the monsoon (July–October) and cool (November–February) seasons. However, during the study period, there was a logging ban countrywide for 2016 and part of 2017. The resting period corresponds to the hot season (March–June), when elephant thermoregulation is compromised due to their small surface-to-volume ratio and difficulty in losing heat (Weissenböck *et al.*, 2011)—when survival is the lowest and calf mortality is the highest (Mumby *et al.*, 2013a,b).

### Study sample and data selection

We collected seasonal health parameters longitudinally from March 2016 to November 2018 for 225 elephants (females, 139; males, 86) aged 4–72 years. We sampled the elephants from three logging regions in the Sagaing Division in Myanmar, namely Kawlin (524 samples), East Katha (176 samples) and West Katha (98 samples). Samples were collected from each individual once in each of the different seasons: the hot season in March–April (283 samples), the monsoon season in July (244 samples) and the cold season in November (271 samples). Animal ethics was approved by MTE, Ministry of Natural Resources and Environmental Conservation in Myanmar and Turku University in Finland for manipulation and sample collection from these animals. The animals used in this study did not present signs of a clinical disease. Despite efforts to sample the same elephants every season, this was sometimes impossible due to translocation or death, but overall, we sampled 5 individuals across 9 seasons, 13 individuals across 8 seasons, 17 individuals across 7 seasons, 22 individuals across 6 seasons, 19 individuals across 5 seasons, 17 individuals across 4 seasons, 24 individuals across 3 seasons, 52 individuals across 2 seasons and, finally, 56 individuals in 1 season only.

### Health measurement

We measured a set of 23 health parameters in order to understand the physiological responses to environmental cues in different seasons. All elephants were measured and sampled during the morning of non-workdays (for details, see Franco dos Santos *et al.*, 2020).

Analysing haematological and serum chemistry parameters (see below for details) required the collection of blood from an ear vein using a Vacuette® system (Greiner Bio-One, Kremsmünster 4550, Austria) with three different tubes, namely ethylenediaminetetraacetic acid (EDTA, 8 ml), heparin (2 ml) and serum separator tubes (18 ml). This was done by trained local veterinarians as part of their regular health monitoring of the animals, in accordance with the local and University of Turku ethical guidelines. The blood tubes were refrigerated for a maximum of 24 hours before analysis in the laboratory. For serum chemistry, the samples were centrifuged (RCF—1320 g) for 20 minutes between 3 and 6 hours after collection and sera was collected and frozen at −20°C. These samples were stored between 6 and 316 days until analysis in a laboratory in Yangon using the IDEXX VetTest® (IDEXX, Westbrook 04092, USA). Several steps were taken to guarantee quality control in serum chemistry analysis, namely (i) the validity of every batch was always confirmed; (ii) some cartridges from the new batches were randomly selected and ran with a sample from the day before, with a maximum of 10% difference accepted; (iii) when a suspicious pattern was observed, a calibration run was performed and the samples reran thereafter; and (iv) once a month, a quality control was performed using pooled samples. These pooled samples were aliquoted and stored in −20°C. Each quality control run was not expected to differ by more than 10% from the first run.

The blood samples collected in EDTA were used to perform a manual count of white blood cells using Turk’s solution (Bjorner and Zhu, 2019). A 100-cell differential leucocyte counts (DWBC) was performed manually using a blood smear stained with Romanowsky solutions (Blumenreich, 1990).

We also measured haematocrit, haemoglobin, sodium, potassium and chloride levels. These data were obtained using a VetScan i-Stat® 1 machine (Abaxis, Union City 94587, USA) with an E3+ cartridge. This device is partially validated for Asian elephants (Tarbert *et al.*, 2017).

Blood pressure was measured using the Omron M6 Comfort IT (Omron, Kyoto 617-0002, Japan) blood pressure monitor with an Intelli wrap cuff (Franco dos Santos *et al.*, 2020), which was applied under the anal skin lap in an area where the diameter of the tail was constant. The elephants were trained by their mahouts to accept tail handling and to keep their tail still.

However, not all health parameters were determined in all samples (missing values: haemoglobin, 195; haematocrit, 170; DWBC, 129; K, 187; Cl, 188; Na, 187) due to changes in methodology and variation in field conditions across our long study period.

### Statistical analysis

To investigate the influence of the three seasons (hot, monsoon and cold) on health parameters, we used two complementary methods: a multivariate joint analysis of all health parameters and a univariate trait-by-trait analysis (referred to as single-trait analysis hereafter). The multivariate analysis allowed us to capture an overall association between season and health by taking into account the covariation of health parameters. In the single-trait analysis, we analysed each health parameter separately in order to describe a specific, potentially seasonal variation in physiology.

#### Clinical chemistry measure and repeatability

We tested the repeatability of our serum chemistry measures analysed using the IDEXX VetTest® analyser by replicating measurements for a subset of individuals (N = 28, technical replicates only). Duplicates were tested after analysing all the 28 samples, using the same operator and dry slides from the same lot. Repeatability was assessed by variance decomposition using the R package *rptR* (Stoffel *et al.*, 2017). For serum chemistry, one individual had abnormally low measures (outside viable range) for Ca, BUN and CK in one replicate, hinting to an analyser malfunction, so we did not include data for this individual.

Repeatability tests emphasized artefactual extreme values due to analyser failures in serum chemistry tests. As a consequence, we removed extreme health parameter values using Horn’s method (Finnegan, 2014) (see Appendix S1). This method determines outliers in a Box-Cox transformed dataset using Tukey’s interquartile (IQR) fences. A data point was considered as an outlier when the point lay 1.5 * IQR outside of the first or third quartile point. Results were qualitatively the same with and without the outliers (Table Appendix 1).

#### Association between seasons and overall health: multivariate mixed procedure

We proceeded in two steps to investigate seasonal variation in health by taking into account the expected correlation between health measures. First, we used a Bayesian multivariate mixed model (package MCMCglmm by Hadfield, 2010) implemented in a framework using Markov chain Monte Carlo (MCMC) sampling. We used a Gaussian error structure and identity link. MCMCglmm uses inverse-Wishart distributed priors for variances. Here, we specified proper priors with parameter ‘V’ for the variances in Res (matrix containing the residual covariances) and in ID (matrix denoting the between individual covariance) set at the repeatability for each trait (Brommer *et al.*, 2014). The parameter ‘nu’ (degree of belief) was equal to the number of health parameters to be estimated in Res and ID (see Brommer *et al.*, 2014).

This model allowed us to include multiple response variables: haematology [TWBC, haematocrit, the absolute count of lymphocytes, monocytes, heterophils and eosinophils measured as (white blood cell/100) * TWBC], serum chemistry (albumin, globulin, ALKP, CK, triglycerides, creatinine and BUN). We reran the multivariate analysis on a second model after removing TWBC and adding the absolute counts from the DWBC. We decided to build two different models because the sum of the absolute counts in the DWBC is a proxy for the TWBC, and they therefore cannot be included in the same model. Because of a large number of missing values, we removed five health responses from the multivariate mixed model (number of missing values out of 798 individual measures: haemoglobin, 195; basophils, 129; K, 187; Cl, 188; Na, 187). We also removed calcium, total proteins and AST from the analyses because of the low repeatability discussed above. We also removed globulins because they are calculated based on total protein values. We included age, sex, region (Kawlin, East and West Katha) and birth origin (wild or captive) as predictors and fitted covariance within individuals by including individual as a random effect. We checked whether our results were dominated by the priors through visual evaluations of the posterior distributions.

We extracted the residuals of the multivariate Gaussian model—our health parameter linearly corrected for the effects of age, sex, region and birth origin. We used a linear discriminant analysis (LDA) in order to assess between-individual variation in health depending on the seasons: discriminant functions are constructed as linear combinations of corrected health estimators that maximize the variance between seasons. The importance of the contribution of each health parameter to these discriminant functions can therefore be interpreted as a measure of how much this parameter varies between seasons, while accounting for the high correlation between health parameters. We tested for the significance of the discriminant values (eigenvalues) using a multivariate analysis of variance (MANOVA) with a Pillai test. The LDA was performed with the ‘ade4’ R package and the function ‘discrimin’ (Dray and Dufour, 2007).

#### Association between seasons and differential blood cell count: multivariate procedure

As the differential blood cell count was performed using a manual approach where a fixed total of 100 cells was counted, errors are necessarily correlated between the different cell types. We analysed seasonal variation in white blood cells types (lymphocytes, monocytes, heterophils, basophiles and eosinophils) using a multivariate generalized linear model framework with a Dirichlet distribution, which allowed the inclusion of multiple response variables (*MGLMreg* from the package MGLM). Because of implementation limitations, we were not able to include random intercepts in this model, so pseudo-replication may be a possible confounding factor in this case. Season of sampling, age, sex, origin (captive or wild-caught) and region were included as explanatory variables.

**Figure 1:**
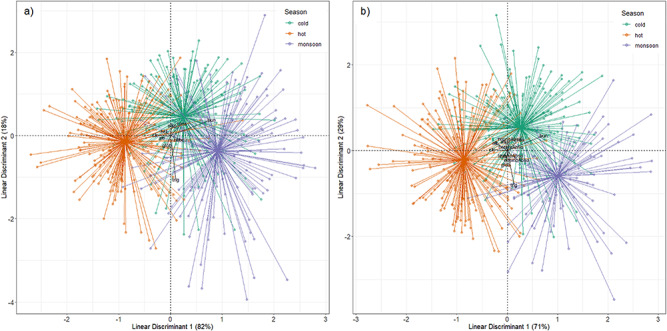
(**A**) Biplot from the LDA of the model with TWBC showing clustering of the ‘hot’, ‘cold’ and ‘monsoon’ seasons across nine health parameters. (**B**) Biplot from the LDA of the model with DWBC showing clustering of the ‘hot’, ‘cold’ and ‘monsoon’ seasons across 12 health parameters. The figure combines the standardized coefficients of health in black (i.e. canonical weights of the linear discriminant functions on the two axes of the LDA) and the projection of the health samples with gravity centres of each season (‘cold’ in blue, ‘hot’ in orange and ‘monsoon’ in violet).

#### Season-related patterns of health parameters: single-trait analysis

Next, we investigated how season was associated with each of the 23 health parameters, independently of the variation in other parameters—a less realistic approach, but one that has the advantage of being robust to possible measurement errors in individual parameters and able to pinpoint specific parameters associated with seasonal variation. Using linear mixed models (*lmer*, package lme4; Bates *et al.*, 2015), we fitted TWBC, haematocrit, haemoglobin, total protein, globulins, albumin, BUN, creatinine, chloride, sodium, potassium, calcium, systolic pressure and diastolic pressure as dependent variables with a Gaussian distribution. Using generalized mixed models (*glmer*, package lme4), we fitted ALKP and CK as dependent variables using a Poisson error distribution with a log link. Using generalized mixed models (*glmmTMB*, package glmmTMB by Mollie *et al.*, 2017), we fitted AST and triglycerides as dependent variables using a zero-inflated negative-binomial error distribution. Our main predictor variable of interest was the three-level season variable fitted in each model as a fixed factor. Our models also controlled for any differences in the health parameters due to age, sex, origin and location. For serum chemistry parameters, serum storage time was also used as a fixed factor. We included individual identity as a random intercept to account for repeated measures from the same animals.

We performed an Akaike information criterion (AIC)-based model selection. For each health parameter, we compared (i) a first model including the confounding variables, (ii) a second model including the confounding variables and the season of sampling and (iii) a third model including the confounding variables and an interaction between the season of sampling and the sex of the elephant (to test whether the health of males and females responds differently to seasonal variation). The most likely models were selected using AIC, considering each random effect as one parameter (Pinheiro, 2002) and selecting the model with the lowest AIC as the best model. Where the difference in AIC between competing models was less than two, we retained the simplest model (Burnham and Anderson, 2002). Akaike weight was also calculated for each model to provide the relative likelihood that it was the best among the candidate models. Further details about model selection and results are presented in Appendix S2.

## Results

### Repeatability of our measurements

Serum chemistry parameters as measured on the IDEXX VetTest® analyser were robust. We showed a high repeatability (>0.9) of BUN, CREA, TRIG and CK and moderately high repeatability (0.6–0.8) of ALB, GLOB and ALKP, but rather low repeatability (<0.6) for Ca, TP and AST.

### Global analysis

The overall health of elephants in both LDA models measuring immune functions—either by the total white cell count (dfhealth = 628 dfseason = 2, Pillai = 0.652, F = 33.36, *P* < 0.001) or by the relative abundance of different types of white blood cells (dfhealth = 537, dfseason = 2, Pillai = 0.744, F = 26.03, *P* < 0.001)—were significantly affected by seasonality. According to the LDA, linear discriminant 1 (TWBC: 82%; DWBC: 71%) clustered the three seasons from hot, cold to monsoon based on measures of 15 health parameters, which can be interpreted as an axis of precipitation/humidity. Linear discriminant 2 (TWBC: 18%; DWBC: 29%) clustered the cold season (green in Fig. 1) from the hot and monsoon seasons (orange and violet, respectively, in Fig. 1), which can be interpreted as an axis of temperature. The discrimination between seasons was particularly higher for three nutritionally driven parameters, BUN, creatinine and triglycerides as well as a muscle marker (CK) that showed the highest loadings (Table 1). These results suggest that body condition and muscle activity are influenced by seasonal variation. These results are not confounded by sampling variation between seasons according to host sex, age, origin or location of sample collection, or the fact that we sampled the same individual several times, as all of these were controlled for in the analysis.

**Table 1:** Standardized coefficients for the health parameters of LD1 and LD2 for the model with TWBC and the model with DWBC

	Model with TWBC		Model with DWBC	
Health parameters	LD1	LD2	LD1	LD2
TWBC	0.173	−0.090		
Lymphocytes			0.070	−0.112
Monocytes			0.053	0.279
Heterophils			0.079	0.093
Eosinophils			0.187	−0.208
Haematocrit	−0.124	0.102	−0.076	−0.125
Albumin	−0.158	−0.051	−0.230	0.186
Globulins	−0.064	−0.228	−0.021	−0.316
BUN	0.797	0.385	0.724	0.357
Creatinine	0.222	0.297	0.241	0.262
CK	−0.294	0.026	−0.300	0.030
ALKP	0.032	0.250	−0.043	0.221
Triglycerides	0.102	−1.051	0.136	−0.792

Health parameters with the highest loadings are highlighted in grey.

LD1 indicates linear discriminant 1; LD2, linear discriminant 2.

### Single-trait analysis of health parameters

In line with the overall health variation, the single-trait analysis shows that several health parameters quantifying haematology, blood pressure and serum chemistry are significantly changed by seasonal variation (see Appendix S3 for models output).

#### Haematology

**Figure 2:**
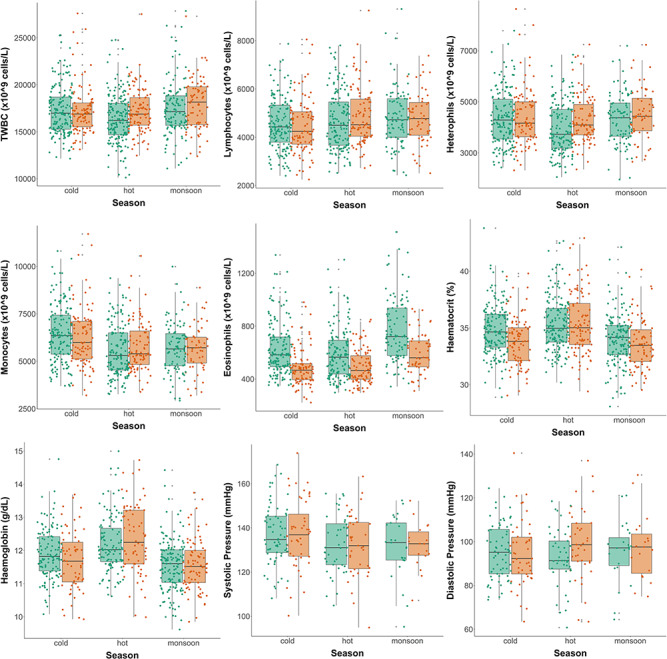
Seasonal changes in haematological and blood pressure parameters in both sexes. Females are represented in green and males in orange. Solid circles represent the unscaled partial residual health traits that account for all main effects (fixed and random confounding variables) but not the effect of sex and season. The horizontal lines within the boxes indicate the medians, boundaries of the boxes indicate the 25th- and 75th-percentiles and the whiskers indicate the highest and lowest values of the results.

On average, elephants had 17 302 × 10^6^ white blood cells/L (TWBC), ranging between 7722 and 29 656 × 10^6^ cells/L across seasons and individuals. Both males and females showed on average lower TWBC in the hot season than in the cold (β = −883.31 ± 331.26, t = −2.667) (Fig. 2) but this variation was more extreme in males. This result was supported by the AIC-model selection that showed that the model including the interaction between season and sex was the best fit to the data (AIC = 14177.68, wAIC = 1; see Table Appendix S2). For the differential white blood cell count that was analysed in a multivariate generalized model, the results varied depending on the cell type considered: basophil numbers were not associated with season, lymphocytes were higher in monsoon than in cold season (β = 0.173 ± 0.085, z = 2.048), heterophils (β = −0.203 ± 0.074, z = −2.726) and monocytes (β = −0.254 ± 0.074, z = −3.431) decreased in the hot season and eosinophils were lower in the hot season (β = −0.172 ± 0.082, z = −2.086) and higher in the monsoon (β = 0.324 ± 0.092, z = 3.542) compared with the cold season (Fig. 2). These results are supported by the AIC-model selection that showed that the model including season as a three-level factor was the best fit to the data (AIC = −12830.25, wAIC = 0.977; see Table Appendix S2).

Red blood cells were similarly associated with seasonal variation. On average, elephants presented 35.1 ± 0.5% haematocrit, with large variance among samples (28–44). In the monsoon season, elephants presented 2.3% less haematocrit (β = −1.030 ± 0.332, t = −3.104) than in the cold season (Fig. 2). AIC-model selection confirmed that the model with an interaction between season as a three-level factor and sex as a two-level factor was the best at explaining variation in haematocrit (AIC = 3010.667, wAIC = 0.760; Table Appendix S2). Although males had lower haematocrit than females in all seasons, this sex difference was largest during the cold season (β = −1.177 ± 0.511, t = −2.304). For haemoglobin levels, on average, elephants presented 11.8 ± 0.2 g/dL, ranging from 9.2 to 16. Compared with the cold season, haemoglobin levels increased by 3.4% in the hot season (β = 0.414 ± 0.101, t = 4.092) and a decrease of 2.5% in the monsoon season (β = −0.282 ± 0.094, t = −3.003) (Fig. 2). This result was supported by the AIC-model selection with the best model including season as a three-level factor (AIC = 1810.522, wAIC = 0.778; Table Appendix S2).

#### Blood pressure

On average, elephants had a systolic pressure of 137 ± 4 mmHg, but there was a large variation within and between individuals (93–172). In both the hot (β = −4.184 ± 3.001, t = −1.394) and monsoon seasons (β = −5.395 ± 3.609, t = −1.495), which both experience high temperatures (dry in the former and with heavy rainfall in the latter), elephants had 2.9% and 3.6% lower systolic pressure than in the cold season, respectively (Fig. 2). Systolic pressure was influenced by an interaction between season as a three-level factor and sex as a two-level factor (AIC = 2002.262, wAIC = 0.952; Table Appendix S2). Females exhibited higher systolic pressure than males in the cold and monsoon seasons but lower systolic pressure in the hot season. On average, elephants had 95 ± 3 mmHg of diastolic pressure, with a large variation among samples (62–140). Similarly to systolic pressure, diastolic pressure was influenced by an interaction between season and sex (AIC = 1975.204, wAIC = 0.980; Table Appendix S2).

#### Protein concentration

On average, elephants had 7.6 ± 0.07 g/dL of total protein, ranging from 6.2 to 9.0, and there was no sex difference (β = −0.097 ± 0.053, t = −1.815). Elephants had on average 3.2 ± 0.03 g/dL albumin (ranging from 2.5 to 3.6) and 4.4 ± 0.05 g/dL globulins (ranging from 3.6 to 5.8). We did not detect any association between protein concentration and season, as the base model was selected as the best model for total proteins, albumin and globulins (AIC = 1009.757, wAIC = 0.998; AIC_albumin_ = 26.544, wAIC_albumin_ = 1; AIC_globulins_ = 479.614, wAIC_globulins_ = 0.999; see Table Appendix S2).

#### Kidney function

**Figure 3:**
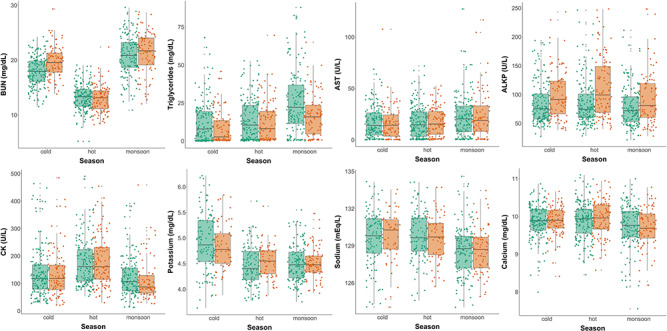
Seasonal changes in kidney function, fat storage, enzymes and electrolytes parameters for both sexes (green for females, orange for males). Solid circles represent the unscaled partial residual health traits that account for all main effects (fixed and random confounding variables) but not the effect of sex and season. The horizontal lines within the boxes indicate the medians of partial residuals, boundaries of the boxes indicate the 25th- and 75th-percentiles and the whiskers indicate the highest and lowest values of the partial residuals.

Elephants had, on average, 19 ± 0.6-mg/dL BUN, ranging from 4 to 34. Season influenced BUN levels (AIC = 4316.746, wAIC = 0.987; Table Appendix S2), which decreased by 29.9% in the hot season compared with the cold season (β = −5.471 ± 0.301, t = −18.161). We observed the opposite pattern in monsoon (with heavy rainfall and good food quality) when BUN increased by 13.6% compared with the cold season (β = 2.496 ± 0.303, t = 8.250) (Fig. 3). On the other hand, creatinine was not affected by season. Elephants displayed creatinine levels of 1.1 ± 0.03 mg/dL on average (sample variance of 0.6–1.7). This result was supported by AIC-model selection in which the base model presented the best AIC (AIC = −132.862, wAIC = 0.342; Table Appendix S2).

#### Circulating fat

Circulating fat was measured using triglyceride level, and elephants presented 11 ± 1.1 mg/dL of triglycerides on average, with large variation between and within individuals (1 to 88). Season played a key role in regulating triglycerides levels, with an increase of 7.9% and 21.8% in the hot and monsoon seasons, respectively, compared with the cold season (β_hot_ = 0.233 ± 0.079, t = 2.969; β_monsoon_ = 0.642 ± 0.073, t = 8.771) (Fig. 3). Males had lower triglyceride levels than females (β = −0.225 ± 0.086, t = −2.619). This result was supported by AIC-model selection with the best model including season as a three-level factor (AIC = 5334.401, wAIC = 0.662; Table Appendix S2).

#### Enzymes

We characterized the liver damage using two enzymes, AST and ALKP, and for the muscular damage, CK. The AST activity displayed by elephants was 29 ± 1 U/L on average, with a large variation between individuals and samples (0–128). The best AST model selected was the model with season as a three-level factor (AIC = 5856.256, wAIC = 0.861; Table Appendix S2). We showed that AST was 8.9% higher in the monsoon than the cold season (β = 0.298 ± 0.065, z = 4.590; Fig. 3). Elephants displayed an average of 114 ± 1 U/L ALKP (ranging from 20 to 249), and similarly to AST, we observed that variation existed between seasons. The model with an interaction between sex and season was retained as the best model (AIC = 7334.816, wAIC = 0.792; Table Appendix S2). Males in the hot season showed higher ALKP levels than females in the cold season (β = 0.104 ± 0.052, z = 1.974), and males had higher ALKP compared with females in the hot (β = 0.120 ± 0.046, z = 2.622) but no difference in the monsoon and cold seasons. With an average of 136 ± 1 U/L ranging from 11 to 491, CK had an increase of 9.3% in the hot season (β = 0.447 ± 0.047, z = 9.559) compared with the cold season (Fig. 3). Similarly to AST, the best model retained for CK had season as a three-level factor (AIC = 8673.050, wAIC = 0.824; Table Appendix S2).

#### Electrolytes

To test whether season could influence electrolyte levels, we measured potassium (average 4.89 ± 0.06 mEq/L, ranging from 3.8 to 6.4), sodium (average 129.68 ± 0.34 mEq/L, ranging from 123 to 134), chloride (average 91.8 ± 0.3 mEq/L, ranging from 83 to 97) and calcium (average 10.0 ± 0.1 mg/dL, ranging from 7.8 to 11.2). We observed an effect of season on sodium levels, with elephants in the monsoon season showing lower levels of this electrolyte compared with the cold season (β = −1.025 ± 0.217, t = −4.727) (Fig. 3). We also observed a higher calcium level in the hot season (β = 0.098 ± 0.046, t = 2.148), compared with the cold season (Fig. 3). We found a lower potassium level in the monsoon season (β = −0.038 ± 0.041, t = −9.320), and this low level was also observed in the hot season compared with the cold season (β = −0.396 ± 0.044, t = −9.078) (Fig. 3). These results were supported by the AIC-model selections of sodium, calcium and potassium, which retained the models including season as a three-level factor as the best models (AIC_sodium_ = 2667.559, wAIC_sodium_ = 0.834; AIC_calcium_ = 1297.149, wAIC_calcium_ = 0.759; AIC_potassium_ = 711.063, wAIC_potassium_ = 0.969; see Table Appendix S2). No seasonal differences were observed for chloride (AIC = 2771.599, wAIC = 0.810; see Table Appendix S2).

## Discussion

Environmental factors varying seasonally such as temperature (Pörtner and Farrell, 2008), food abundance (Hanya *et al.*, 2006) or rainfall (Strong and Sherry, 2000) can have both direct and indirect effects on animal physiological processes. Theoretically, long-lived species are often predicted to be buffered against an environmental heterogeneity: longevity means low annual mortality and reproduction rates and annual variability in climate may therefore have a smaller impact on the population growth rates of long-lived species compared with short-lived ones (Morris *et al.*, 2008). Nevertheless, a longitudinal study of wild African elephants in Amboseli, Kenya, found that drought years were associated with higher calf mortality (Moss, 2001; Foley *et al.*, 2008). Similarly, warm, wet and windy winter conditions were associated with reduced juvenile survival but increased adult fecundity and survival in Soay sheep (Forchhammer *et al.*, 2001), but little is known of the physiological mechanisms for such patterns. In this study, we investigated seasonal variation in the health of Asian elephants occupying tropical forests with a strong seasonal monsoon climate (Beck *et al.*, 2018). As we sampled a large number of animals with repeated measures of several health parameters, we were able to demonstrate both global and individual trait-by-trait seasonal effects on health. The global analysis highlighted the biggest differences in health between the hot and monsoon seasons. Previous studies have found Asian elephants to display higher survival in months with intermediate temperatures and more rainfall (Mumby *et al.*, 2013b), and our results may offer some mediating mechanisms for such seasonally increased mortality risk in juveniles and decreased survival chance for elephants of all ages (Mumby *et al.*, 2013a,b). Importantly, adult elephants aged between 18 and 50 years work in our study population (Tun Aung and Thoung Nyunt, 2001). However, during the study period, there was a logging ban countrywide for 2016 and part of 2017. Given the working age, elephants represent only 25% of the total samples and 79% of those are females with a vast majority being pregnant or mothers with calves younger than 2 years old (Crawley *et al.*, 2020) and thus not subject to workload; seasonal workload patterns are very unlikely to drive our results. More generally, the results suggest that even long-lived, large mammals that are generally thought to be well equipped against climate variation may experience significant physiological changes in response to regular seasonal variation in their environment that can, in extreme circumstances, pose a risk.

We measured altogether 23 health monitoring parameters related to haematology, blood pressure, liver, muscular and kidney function, electrolytes and circulating fat. While each parameter was informative of the underlying ecophysiology in their own right, we also developed an innovative approach to take into account the interdependency of the overlapping physiological functions (Trayhurn, 2005). The use of a multivariate method to establish the difference between the hot and monsoon seasons offered the advantage of detecting and accounting for the interdependency between health parameters, which is not possible when analysing individual traits. Such a global approach revealed that season was a major driver of health, especially during periods characterized by the extremes of rainfall, namely the hot season with no rainfall and the monsoon season with heavy rainfall. Seasonality has a strong effect on the growth and production of vegetation, with rainfall being one of the most important factors in predicting vegetation mass (Austin, 2002; Arndal *et al.*, 2009). In both of our overall health analyses, the health factors that had the biggest contribution to the overall health variation across seasons were the three parameters related to nutrition. Firstly, triglyceride levels, which indicate nutritional status (Morfeld *et al.*, 2016), contributed significantly to the seasonal variation in overall health, with the lowest circulating fat levels apparent in the cold season and the highest levels during the monsoon season. Secondly, BUN, also a main driver in health variation with season, had its lowest value in the hot season and its highest in the monsoon season. The third factor that has influence in both models is CK. This enzyme reflects muscle damage and it reaches a peak in the hot season and its lowest in the monsoon season. These may reflect food quality and quantity fluctuations across the year: during the hot and dry seasons, Asian elephants are largely browsers, while in the monsoon season, they are grazers and may eat more fruit (Sukumar, 2003). This influences their protein intake, since in the dry season, elephants ingest low levels of protein (browse period), and in wet seasons, they consume higher protein levels (grazing periods) (McCullagh, 1969; Brown and White, 1979). A study assessing the protein content of the favourite food plants consumed by our study elephants in Myanmar across different seasons also found that the crude protein was significantly higher in wet versus dry season plants (Dierenfeld *et al.*, 2020). Such foraging differences in our study elephants are especially detectable in their BUN levels, with BUN being the main nitrogenous end product of protein metabolism (Fowler and Mikota, 2006).

The hot season is characterized by high temperatures, no rainfall and consequently, low food quality and quantity as well as lower water availability. In the MTE elephant population, the hot season also corresponds to the resting period when elephants do not work. During this season, we observed a peak in haemoglobin; an increase observed from the monsoon until the hot season, possibly due to variation in nutritional condition and food intake; and the haemoconcentration attributed to dehydration (DelGiudice *et al.*, 1992; Hellgren *et al.*, 1993). CK also reached the highest level in the hot season. As many of our sample elephants worked little or not at all during the study period, due to the current logging ban, the CK variation we observed may reflect a natural seasonal variation in physiological processes rather than workload. Physical activity and an increase in respiration rate at high temperatures can cause CK to increase (Chulayo and Muchenje, 2013). We also found that blood pressure, BUN and creatinine displayed their lowest levels in hot season. Blood pressure is known in humans to be affected by seasonality (Rosenthal, 2004; Lewington *et al.*, 2012), and in our study, males had higher blood pressure than females in the hot season. High temperatures cause increased vasodilatation (widening of the arteries and veins) and loss of water and salt through sweating, leading to lower blood pressure (Rosenthal, 2004). Elephants have difficulty sweating and losing heat (Hiley, 1975), so this decline in blood pressure during hot months was likely produced by increased vasodilatation. BUN levels were lowest during the hot season, alongside lowest food availability, indicating that protein metabolism in each season may play a stronger role in influencing these parameters than the glomerular filtration rates from the kidneys (DelGiudice *et al.*, 1992; Crooks *et al.*, 2000; Butler *et al.*, 2006).

Unlike the hot season, the monsoon season is characterized by strong and prolonged precipitation associated with high temperatures, as well as increased food quality, food quantity and ease of access to water. We observed higher values in the monsoon season for the different white blood cells and they were also maintained at high levels during the cold season (except for lymphocytes) compared with the hot season. Such increases may be driven by infectious diseases given that infectious agents require specific climatic conditions to spread and infect their hosts, and the hosts in turn may present different resistance to their infection during different seasons (Hosseini *et al.*, 2004; Altizer *et al.*, 2006). The host immune system can adapt to such seasonality in infection and be more active in times when the conditions for the infectious agents are more prevalent (Hosseini *et al.*, 2004). In line with this, the levels of the gastrointestinal parasites prevalent in our study population begin to rise in June from low levels during the hot season, until the peak levels recorded in December/January of the cold season (Lynsdale, 2017). Another health parameter to rise during the monsoon was AST, which is present in several tissues, especially in the liver, cardiac muscle and skeletal muscle. The observed AST rise in the monsoon season here may be an indicator of an increased liver damage, as there is often also a decrease in CK in this season that is more sensitive to muscular damage than AST (Fowler and Mikota, 2006). The cause behind the rise in AST during the monsoon season is not clear to us at present, as ALKP, another liver enzyme, peaks in the hot season rather than the monsoon. BUN also increased in the monsoon season, and as stated previously, may be explained more by nutritional status and by glomerular filtration in the kidneys (DelGiudice *et al.*, 1992; Crooks *et al.*, 2000; Butler *et al.*, 2006). We also observed that male BUN levels were higher than those of females in the monsoon and cold seasons. Conversely, haematocrit and haemoglobin had their lowest values during this season. Males normally have higher levels in haematocrit and haemoglobin due to the positive effect of testosterone on erythropoiesis (Riviello and Wirz, 2001; Howell *et al.*, 2003), but for reasons still unknown to us, we found that haematocrit and haemoglobin presented no difference between sexes.

The cold season is characterized by low temperatures with a lower rate of rainfall and a better quality and quantity of food at the beginning of the season, decreasing in the run up to the hot season. In this season, there is a peak in gastrointestinal parasite abundance (Lynsdale, 2017). The total and different white blood cells remained higher compared with the hot season (except for lymphocytes that decreased) with reasons that can range from possible circulating infectious agents such as pathogenic bacteria and virus, nutritional status and photoperiod. Immune system activation and maintenance exert energy costs on an individual, and therefore, it would be expected that animals with a better body condition have more active immune systems (Gilot-Fromont *et al.*, 2012; Warburton *et al.*, 2016). In addition to this, decreases in the photoperiod have been shown to have a positive effect on the ability of the immune response to anticipate immunological challenges (Nelson, 2004). It is possible that the increase in white blood cells (especially of the innate immune system cells) observed in the monsoon and cold seasons could be related to a higher infection risk, a better body condition and a shorter photoperiod (3-hour difference between the longest and the shortest daylight days). However, this would only be the case in cells that compose the innate immune system (Kantari *et al.*, 2008) since lymphocytes in the cold season decrease to levels comparable to the hot season. Blood pressure (specifically systolic pressure) also peaked during the cold season, with males displaying lower or the same blood pressure as females. The reason may be similar to humans, where cooler temperatures increase the sympathetic nervous system tone, increasing the blood pressure in this way (Rosenthal, 2004).

Studying the seasonal variation of health in natural environments is difficult to conduct in wild populations. Utilizing a semi-captive population such as the MTE timber elephants has numerous advantages. Firstly, these animals preserve many natural behaviours such as roaming, feeding and mating and may carry these out with wild conspecifics and without any human control (Lynsdale *et al.*, 2017). Secondly, these elephants are monitored by trained veterinarians who check their health every month, ensuring the animals can be handled, identified and used for longitudinal health data collection. However, our study does have some limitations that need to be considered. Data collection once every season limits the possibility of observing detailed trends in health from season to season, and investigating how quickly or slowly health changes diminish. Further, few previous studies exist from similar climate systems, preventing us from drawing comparisons as the majority of previous work has been conducted in either (i) continental climate systems (Beck *et al.*, 2018), (ii) with hibernating species (Hissa *et al.*, 1994; Huber *et al.*, 1997; Yang *et al.*, 2018), (iii) with predator species such as wolves (Seal and Mech, 1983; Butler *et al.*, 2006) or (iv) with aquatic species (Trumble *et al.*, 2006; Norman *et al.*, 2012; Yu *et al.*, 2014). This clear gap in our knowledge calls for more research on ecological health variation to be conducted on a wider range of species occupying a range of climatic conditions.

In conclusion, our results demonstrate that season is a major driver of health parameters, with all except 5 of the 23 parameters differing between seasons. Climate change is predicted to increase extreme weather patterns in monsoon climates and could bring major challenges to natural systems, especially for species already endangered, as is the case for Asian elephants. A third of the remaining Asian elephant population lives in captivity, mostly in countries exposed to seasonal monsoon climate, meaning our results also have practical and relevant implications for Asian elephant management and highlight the importance of considering seasonal health variation when assessing veterinary care and workload decisions.

## Funding

This work was supported by the European Research Council (ERC-2014-CoG 648766), the Academy of Finland (324257), the Turku Collegium for Science and Medicine, Marie Sklodowska-Curie (659937—AGEISM and 794087—GAE) and the University of Sheffield.

## Author Contributions

Diogo Franco dos Santos, Sophie Reichert and Virpi Lummaa conceived the ideas and designed methodology; Diogo Franco dos Santos, Robin Cristofari, Win Htut, Htoo Htoo Aung and U Kyaw Nyein collected the data; Diogo Franco dos Santos, Vérane Berger and Robin Cristofari analysed the data; Diogo Franco dos Santos, Vérane Berger, Sophie Reichert and Virpi Lummaa led the writing of the manuscript. All authors contributed critically to the drafts and gave final approval for publication.
